# Roles of fibroblast growth factor 21 in the control of depression‐like behaviours after social defeat stress in male rodents

**DOI:** 10.1111/jne.13026

**Published:** 2021-09-01

**Authors:** Naoki Usui, Masahide Yoshida, Yuki Takayanagi, Naranbat Nasanbuyan, Ayumu Inutsuka, Hiroshi Kurosu, Hiroaki Mizukami, Yoshiyuki Mori, Makoto Kuro‐o, Tatsushi Onaka

**Affiliations:** ^1^ Division of Brain and Neurophysiology Department of Physiology Jichi Medical University Shimotsuke Japan; ^2^ Department of Dentistry, Oral and Maxillofacial Surgery Jichi Medical University Shimotsuke Japan; ^3^ Division of Anti‐aging Medicine Center for Molecular Medicine Jichi Medical University Shimotsuke Japan; ^4^ Division of Genetic Therapeutics Center for Molecular Medicine Jichi Medical University Shimotsuke Japan

**Keywords:** catecholaminergic neurones, depression‐like behaviour, FGF21, nucleus tractus solitarius, social defeat stress

## Abstract

Fibroblast growth factor 21 (FGF21) modulates energy metabolism and neuroendocrine stress responses. FGF21 synthesis is increased after environmental or metabolic challenges. Detailed roles of FGF21 in the control of behavioural disturbances under stressful conditions remain to be clarified. Here, we examined the roles of FGF21 in the control of behavioural changes after social defeat stress in male rodents. Central administration of FGF21 increased the number of tyrosine hydroxylase‐positive catecholaminergic cells expressing c‐Fos protein, an activity marker of neurones, in the nucleus tractus solitarius and area postrema. Double in situ hybridisation showed that some catecholaminergic neurones in the dorsal medulla oblongata expressed β‐Klotho, an essential co‐receptor for FGF21, in male mice. Social defeat stress increased FGF21 concentrations in the plasma of male mice. FGF21‐deficient male mice showed social avoidance in a social avoidance test with C57BL/6J mice (background strain of FGF21‐deficient mice) and augmented immobility behaviour in a forced swimming test after social defeat stress. On the other hand, overexpression of FGF21 by adeno‐associated virus vectors did not significantly change behaviours either in wild‐type male mice or FGF21‐deficient male mice. The present data are consistent with the view that endogenous FGF21, possibly during the developmental period, has an inhibitory action on stress‐induced depression‐like behaviour in male rodents.

## Graphical abstract

Social defeat stress increased plasma concentrations of FGF21. Catecholaminergic neurones in the nucleus tractus solitarius and area postrema expressed β‐Klotho, an essential co‐receptor for FGF21, and were activated by social defeat stress and FGF21 administration. FGF21‐deficient mice showed augmented depression‐related behaviours after social defeat stress.

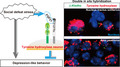

## 1 INTRODUCTION

Fibroblast growth factor 21 (FGF21) is synthesised in several organs including the liver, pancreas, muscle and brain.^1^, ^2^, ^3^, ^4^, ^5^, ^6^, ^7^ Nutritional or environmental challenges, including food deprivation, overfeeding, ketogenic or high‐carbohydrate diet, and physical exercise, have been shown to increase the expression of FGF21 in peripheral organs and/or in the brain.^8^ FGF21 acts on receptor complexes consisting of the FGF receptor (FGFR1c, FGFR2c or FGFR3c) and β‐Klotho.^9^, ^10^, ^11^ FGF21 has been shown to induce a shift to catabolic metabolism including glucose uptake and fatty acid oxidation in white adipose tissue, fatty acid β‐oxidation and reduction of triglyceride accumulation in the liver, and an increase in energy expenditure.^3^

Within the brain, β‐Klotho, which is essential for high affinity binding of FGF receptors to FGF21, has been shown to reside in the hypothalamus, area postrema and nucleus tractus solitarius, whereas FGF receptors are broadly expressed in the brain.^12^ Brain regions where β‐Klotho resides play an important role for maintenance of homeostasis under stressful environmental challenges by controlling behavioural and autonomic responses. Indeed, FGF21 has been shown not only to control energy homeostasis, but also to modulate stress responses and emotional behaviours. FGF21 activates the sympathetic nervous system and the hypothalamic‐pituitary adrenal axis.^4^, ^12^ High serum levels of FGF21 were reported in bipolar manic patients.^13^ FGF21 levels in depressed bipolar disorder patients have been shown to be affected by mood stabiliser treatments.^13^, ^14^ A negative correlation between cerebrospinal fluid FGF21 level and depression scores has also been reported.^15^ Furthermore, administration of FGF21 has been reported to reduce lipopolysaccharide‐induced depression‐like behaviours in mice,^16^ although FGF21 has also been reported to induce anxiety‐related behaviour.^17^, ^18^ It remains unclear whether endogenous or exogenous FGF21 modulates psychological stress‐induced depression‐like behaviours.

Social defeat stress, which is an ethologically relevant social stress, has been used as an animal model for induction of depression‐like behaviours in mice.^19^, ^20^ Depression has been shown to be associated with low activity of noradrenergic neurones in the brain,^21^ whereas activation of noradrenergic neurones has been shown to attenuate depression‐like behaviours observed after social defeat stress.^22^ On the other hand, selective destruction of noradrenergic neurones in the nucleus tractus solitarius has been reported to augment depression‐like behaviour,^23^ suggesting anti‐depressive action of noradrenergic neurones in the nucleus tractus solitarius. β‐Klotho is expressed in the nucleus tractus solitarius. Our previous study showed that i.c.v. injection of FGF21 activated cells in the nucleus tractus solitarius in rats.^24^ Using these brains, we first determined whether central administration of FGF21 activated catecholaminergic neurones in the nucleus tractus solitarius by examining the expression of c‐Fos protein in tyrosine hydroxylase‐positive neurones in the medulla oblongata. We next determined whether tyrosine hydroxylase‐positive neurones in the nucleus tractus solitarius expressed *β‐Klotho* mRNA using double in situ hybridisation. We also determined whether social defeat stress increased FGF21 concentrations in plasma and activated catecholaminergic neurones in the nucleus tractus solitarius. We then examined whether endogenous FGF21 modulated depression‐like behaviours by analysing social defeat stress‐induced depression‐like behaviours in FGF21‐deficient mice. We further examined the effects of overexpression of FGF21 using adeno‐associated virus (AAV) vectors on depression‐like behaviours in wild‐type and FGF21‐deficient mice.

## 2 MATERIALS AND METHODS

### 2.1 Animals

Male C57BL/6J mice (CLEA Japan, Shiga, Japan), retired CD1mice (Charles River Laboratories Japan, Kanagawa, Japan) and FGF21‐deficient mice (C57BL/6J background)^25^ were used in the present study. Male Wistar rats (Slc:Wistar; Japan SLC, Inc., Shizuoka, Japan) were used for i.c.v. injection of FGF21.

The animals were housed in rooms under a 12:12‐hour light/dark photocycle (lights on 7:30 am) at 20‐24℃ and 40%‐70% relative humidity. Food and water were available ad lib. All animal procedures were approved by the Institutional Animal Experiment Committee of Jichi Medical University and were in accordance with the Institutional Regulations for Animal Experiments and Fundamental Guidelines for Proper Conduct of Animal Experiments and Related Activities in Academic Research Institutions under the jurisdiction of the Ministry of Education, Culture, Sports, Science and Technology.

### 2.2 Intracerebroventricular injections of FGF21

Rats were anaesthetised with an i.p. injection of 200 mg kg^‐1^ tribromoethanol (Avertin; Wako Pure Chemical Industries, Ltd, Osaka, Japan) and placed in a stereotactic frame. A stainless steel guide cannula of 23 gauge was inserted into the right lateral cerebral ventricle at coordinates of 0.6 mm caudal to the bregma, 1.6 mm lateral to the midline and 4.5 mm below the skull. The rats were allowed to recover in individual cages for 1‐2 weeks and were given an i.c.v. injection of mouse FGF21 at doses of 0, 10, 100 and 1000 ng per 5 μL with a 30‐gauge needle. The vehicle consisted of 147 mmol L^‐1^ sodium chloride, 1.2 mmol L^‐1^ calcium chloride, 0.9 mmol L^‐1^ magnesium chloride, 4 mmol L^‐1^ potassium chloride, 1.8 mmol L^‐1^ HEPES (pH 7.4) and 0.1% rat serum albumin. Recombinant FGF21 was a generous gift from Dr Moosa Mohammadi (New York University School of Medicine, New York, NY, USA). Recombinant mouse FGF21 (residues 33‐209) was produced in *Escherichia coli*, refolded in vitro, and purified by affinity, ion exchange and size exclusion chromatography as described previously.^26^ The number of animals injected with 0, 10, 100 and 1000 ng FGF21 was 6, 6, 6 and 5, respectively. The same brains were used in a previous study showing single immunocytochemical detection of c‐Fos protein.^24^

### 2.3 AAV vectors

AAV8 vectors expressing mouse FGF21 or humanised *Renilla reniformis* green fluorescent protein (hrGFP) under the control of the CAG promoter (chicken β‐actin promoter with the cytomegalovirus immediate‐early enhancer) were prepared as described previously.^24^, ^27^ In brief, 293 cells were transfected simultaneously with adenovirus helper plasmid, AAV type 8 helper plasmid, and the hrGFP or FGF21 plasmid by calcium phosphate transfection and were maintained for 3 days. The AAV vectors were purified by cesium chloride density gradient centrifugation and the titres of the AAV vectors were measured by a quantitative real‐time polymerase chain reaction. Each mouse was i.p. injected with AAV‐FGF21 at a dose of 5.0 × 10^8^ or 1.0 × 10^9^ viral genomes (vg) or with 1.0 × 10^9^ vg of AAV‐hrGFP in a volume of 500 μL. Two weeks after injection, behavioural experiments were started. The number of wild‐type mice injected with AAV‐hrGFP, 5.0 × 10^8^ AAV‐FGF21 and 1.0 × 10^9^ AAV‐FGF21 was 12, 13, and 12, respectively. In experiments with FGF21‐deficient mice and AAV‐FGF21, the number of FGF21‐deficient mice injected with AAV‐hrGFP was 10 and the number of FGF21‐deficient mice injected with AAV‐FGF21 was 11.

### 2.4 Social defeat stress

Social defeat stress was applied as described previously.^20^ In brief, aggressive CD1 mice, which were selected as reported previously,^19^ were kept singly in their home cages (each 91 × 260 × 128 mm in size) for more than 2 days. The test mouse was introduced as the intruder into the cage of the aggressive resident male CD1 mouse and kept there for 10 minutes. This procedure of social defeat stress was applied once per day for 5 consecutive days. Behaviours of the intruder mouse during exposure to the resident aggressor mouse were videotaped. The duration of defeat posture (an upright posture with the belly exposed toward the resident), duration of immobility behaviour (no movements except for respiration with four paws on the ground) and duration of escape (rapid escape movements away from the aggressor) were measured in the first and fifth stress sessions of the experiments with FGF21‐deficient mice. A non‐stressed control mouse was placed in an empty cage and was kept there for 10 minutes. This procedure was repeated once per day for 5 days.

Ten animals in each group were used for measurements of plasma concentrations of FGF21. In experiments with FGF 21‐deficient mice and wild‐type mice, the number of wild‐type mice was 6 and the number of FGF21‐deficient mice was 8. In experiments for immunocytochemical detection of c‐Fos protein and tyrosine hydroxylase, social defeat stress was applied once and the number of animals for non‐stress and social defeat stress was 8 and 6, respectively. The same brains were used in a previous study showing immunocytochemical detection of c‐Fos protein and oxytocin.^20^

### 2.5 Behavioural analysis

Behavioural tests were performed before and after social defeat stress in experiments with FGF21‐deficient mice and in experiments with mice injected with AAV‐FGF21. In the experiments with FGF21‐deficient mice, an elevated plus maze test, social avoidance test, tube test, social interaction test and forced swimming test were performed sequentially before and after social defeat stress. In the experiments using mice injected with AAV‐FGF21, an elevated plus maze test, social avoidance test and forced swimming test were performed sequentially before and after social defeat stress. In the experiments using FGF21‐deficient mice injected with AAV‐FGF21, a social avoidance test and a forced swimming test were performed sequentially before and after social defeat stress. The intervals between the behavioural tests were at least one day for each animal. Amounts of water intake per day and food intake per day were recorded by measuring weights of water bottles and remaining food pellets in individual cages in the morning of the day of AAV injection, in the morning of the day of the first session of social defeat stress before the application of social defeat stress and in the next morning after the day of the fifth session of social defeat stress.

#### 2.5.1 Elevated plus maze test

The maze consisted of two enclosed arms (25 × 5 × 15 cm, transparent walls) and two open arms (10 lux; O'Hara & Co., Ltd, Tokyo, Japan). Total locomotion activity, time spent in open arms and frequency of entry into open arms were automatically measured during the 10‐minute test duration using a behaviour analysing program, Time EP1 (O'Hara & Co., Ltd), as described previously.^28^

#### 2.5.2 Social avoidance test

A test mouse was introduced into a test cage (15 × 28.5 × 39.5 cm) and was kept there for 3 minutes as a habituation session. In the test cage, two empty cylinders (each 7 cm in diameter and 16.3 cm in height) were placed at opposite corners of the cage in the habituation session. Each cylinder had multiple holes in the lower part. Three minutes after the habituation session, the test mouse was introduced again into the test cage and kept there for another 3 minutes as a test session. Before the test session, an ICR mouse or C57BL/6J mouse that had no prior contact with the test mouse was placed in one of the cylinders and kept in the cylinder during the test session. Total locomotion activity and duration spent for sniffing perforated parts of the cylinder containing a mouse were measured during the 3‐minute test session using a behaviour analysing program, Time SSI (O'Hara & Co., Ltd).

#### 2.5.3 Social interaction test

In the experiments with FGF21‐deficient mice, a social interaction test was performed to assess social contact in a familiar environment. In the test, social contact between two mice in the test cage was measured with a system that automatically analyses behaviour in cages (each 29 × 18 × 12 cm in size) (O'Hara & Co., Ltd), as described previously.^28^ A wild‐type or FGF21‐deficient mouse was placed in pair with a C57BL/6J mouse in the test cage. Images from each cage were captured at a rate of two frames per second and the number of particles in each frame was counted automatically for 30 hours using behaviour analysing software (Time HC8_Multi; O’Hara & Co., Ltd); two particles indicated that the mice were not in contact with each other and one particle indicated contact between the two mice. Reduction of social contact has been detected in various animal models of psychiatric disorders with impaired social behaviour.^28^, ^29^, ^30^

#### 2.5.4 Forced swimming test

Test mice were individually placed into a cylinder (11 cm in diameter, 25.5 cm in height) containing water at a depth of 15 cm (23℃). Total cumulative time spent for immobility behaviour was determined automatically during the 6‐minute test using behaviour analysing software (Time FZ1; O’Hara & Co., Ltd).

### 2.6 Single in situ hybridisation and double in situ hybridisation for detection of mRNA of *tyrosine hydroxylase* and *β‐Klotho*

Brains of C57BL/6J mice were freshly obtained by decapitation and immediately frozen in powdered dry ice. Fresh frozen sections were cut coronally at intervals of 20 μm with a cryostat. Sections were mounted on silane‐coated glass slides and then processed for mRNA detection as described previously.^31^

Fluorescein‐ or digoxigenin (DIG)‐labelled cRNA probes were prepared to detect single mRNA and multiple mRNAs simultaneously. cDNA fragments of mouse *tyrosine hydroxylase* (1‐1025 bp; GenBank, NM_009377) and mouse *β‐Klotho* (110‐646 bp; GenBank, AF165170^32^) were subcloned into the Bluescript II plasmid vector. In vitro transcription was performed using T3 or T7 RNA polymerase (Roche Diagnostics GmbH, Mannheim, Germany).

Sections were treated at room temperature with 4% paraformaldehyde in 0.1 mol L^‐1^ phosphate buffer (pH 7.4) for 15 minutes, 0.25% acetic anhydride in 0.1 mol L^‐1^ triethanolamine‐HCl (pH 8.0) for 10 minutes and a hybridisation buffer (50% formamide, 50 mmol L^‐1^ Tris‐HCl, pH 7.5, 1 × Denhardt's solution, 0.6 mol L^‐1^ NaCl, 200 μg mL^‐1^ yeast tRNA, 1 mmol L^‐1^ ethylenediaminetetraacetic acid and 10% dextran sulphate) for 30 minutes. Hybridisation was performed at 63.5℃ overnight in the hybridisation buffer supplemented with cRNA probes at a dilution of 1:1000. Post‐hybridised sections were washed at 61℃ with 5 × SSC containing 0.0005% Tween 20 for 30 minutes, 4 × SSC containing 50% formamide and 0.001% Tween 20 for 40 minutes, 2 × SSC containing 50% formamide and 0.001% Tween 20 for 40 minutes, and 0.1× SSC containing 0.0005% Tween 20 for 30 minutes. Then the sections were incubated at room temperature in NTE buffer (0.5 mol L^‐1^ NaCl, 0.01 mol L^‐1^ Tris‐HCl, pH 8.0, 5 mmol L^‐1^ ethylenediaminetetraacetic acid and 0.0005% Tween 20) for 20 minutes, 20 mmol L^‐1^ iodoacetamide in NTE buffer for 20 minutes and TNT buffer (0.15 mol L^‐1^ NaCl, 0.1 mol L^‐1^ Tris‐HCl, pH 7.4, and 0.0005% Tween 20) for 20 minutes. The sections were then incubated at room temperature with DIG blocking solution (1% blocking reagent (Roche Diagnostics GmbH), 10% normal sheep serum in TNT buffer) for 30 minutes and 0.5% Tyramide Signal Amplification (TSA) blocking reagent (PerkinElmer Life and Analytical Sciences, Boston, MA, USA) in TNT buffer for 30 minutes. For detection of the DIG‐labelled cRNA probe (*β‐Klotho*), sections were incubated at room temperature with peroxidase‐conjugated anti‐DIG antibody (dilution 1:500; Roche Diagnostics GmbH; RRID:AB_514500) for 90 minutes followed by visualisation with the Cy3‐TSA plus amplification kit (PerkinElmer Life and Analytical Sciences). For detection of the fluorescein‐labelled cRNA probe (*tyrosine hydroxylase*), sections were incubated at room temperature with peroxidase‐conjugated anti‐fluorescein antibody (dilution 1:500; Roche Diagnostics GmbH; RRID:AB_840257) for 90 minutes followed by visualisation with the Cy5‐TSA plus amplification kit (PerkinElmer Life and Analytical Sciences). Images were taken with a confocal microscope (TCS‐SP5; Leica Microsystems, Wetzlar, Germany). In experiments with double in situ hybridisation, residual activities of peroxidase introduced for detection of *β‐Klotho* mRNA were inactivated by incubation with 1.0% H_2_O_2_ in TNT buffer at room temperature for 30 minutes and sections were processed for detection of *tyrosine hydroxylase* mRNA. The number of sections for detection of *β‐Klotho* and *tyrosine hydroxylase* mRNA in the area postrema and the nucleus tractus solitarius was 3 and 5, respectively in each animal. The number of animals used for single in situ hybridisation was 2 and the number used for double in situ hybridisation was 4.

### 2.7 Measurements of FGF21 and corticosterone

Plasma FGF21 levels were measured using an FGF21 Mouse/Rat ELISA Kit (RD291108200R; BioVendor, Brno, Czech Republic) in accordance with the manufacturer's instructions. Plasma corticosterone concentrations were measured using a Corticosterone ELISA Kit (No. 501320; Cayman Chemical, Ann Arbor, Michigan, USA). In experiments for measurements of plasma hormone concentrations after social defeat stress, mice were subjected to 10‐minute social defeat stress once per day for 4 days and blood samples were obtained by decapitation just before (0 minutes), 30 minutes after, 60 minutes after or 120 minutes after the fifth social defeat stress. Control mice were handled similarly to stressed mice without exposure to social defeat stress. In the experiments with C57BL/6J mice injected with AAV vectors, blood samples were obtained 33 days after injections of AAV vectors for measurements of plasma FGF21 concentrations. In experiments with FGF21‐deficient mice injected with AAV vectors, blood samples were obtained 26 days after injections of AAV vectors.

### 2.8 Immunocytochemical detection of c‐Fos protein, tyrosine hydroxylase and prolactin‐releasing peptide (PrRP)

One hundred minutes after injection of FGF21 or a vehicle, the animals were deeply anesthetised with Avertin (tribromoethanol, 200 mg kg^‐1^ body weight) and perfused with a fixative solution containing 2% paraformaldehyde and 3.75% acrolein in 0.1 mol L^‐1^ phosphate buffer (pH 7.4). In the experiments with mice after social defeat stress, one hundred minutes after the end of 10‐minute social defeat stress, mice were deeply anaesthetised with Avertin (tribromoethanol, 200 mg kg^‐1^ body weight) and perfused with 4% paraformaldehyde followed by heparinised saline.

Brain sections were cut coronally at intervals of 30 μm with a freezing sledge microtome and then processed for immunohistochemical detection of c‐Fos and tyrosine hydroxylase as described previously.^20^, ^33^

In brief, sections were incubated with a rabbit polyclonal antibody against the N‐terminal region (4‐17) of the c‐Fos peptide sequence (dilution 1:10 000; Ab‐5; Oncogene Science Diagnostics, Cambridge, MA, USA; RRID:AB_2314042; at 4℃ for 2 days) and with peroxidase‐labelled goat anti‐rabbit immunoglobulin G (IgG) (dilution 1:500; PI‐1000; Vector Laboratories, Burlingame, CA, USA; at 4℃ overnight). Immunoreactivity of c‐Fos protein was visualised as a black nuclear profile by incubation with 3,3′‐diaminobenzidine tetrahydrochloride (DAB), nickel sulphate, glucose and glucose oxidase.

Sections were then processed for immunocytochemical detection of tyrosine hydroxylase.^33^ Sections were incubated with rabbit anti‐tyrosine hydroxylase antibody (dilution 1:7500; AB152; Chemicon, Temecula, CA, USA; RRID:AB_390204; at 4℃ overnight), biotinylated anti‐rabbit IgG (dilution 1:750; BA1000; Vector Laboratories; at room temperature for 2 hours) and avidin biotinylated horseradish peroxidase complex (dilution 1:50; Elite ABC kit; Vector Laboratories; at room temperature for 30 minutes), followed by DAB solution for visualisation of tyrosine hydroxylase immunoreactivity. The number of sections for detection of c‐Fos protein and tyrosine hydroxylase in the nucleus tractus solitarius and ventrolateral medulla was 13 or 10 in each rat and mouse. The number of sections counted for the area postrema was 3 for each rat and 3 for each mouse. The intervals between sections were 120 μm for rats and 90 μm for mice.

Immunocytochemical detection of PrRP was performed as described previously.^34^ In brief, sections were incubated with a mouse anti‐PrRP monoclonal antibody (dilution 1:1600; P2L‐1T; Takeda Pharmaceutical Company, Osaka, Japan; RRID:AB_2883968; at 4℃ for 2 days), biotinylated anti‐mouse IgG (dilution 1:500; BA2001; Vector Laboratories; at room temperature for 2 hours) and avidin biotinylated horseradish peroxidase complex (dilution 1:50; ABC kit; Vector Laboratories; at room temperature for 30 minutes), followed by DAB solution for visualisation of PrRP immunoreactivity. The number of sections was 7 for each animal.

Pre‐absorption of the c‐Fos antibody with the synthetic peptide human c‐Fos (amino acid residues 4‐17 of human c‐Fos) blocked immunostaining^35^ and brain sections of c‐Fos‐deficient mice showed no immunoreactive staining.^36^ Specificity of the PrRP antibody was demonstrated by a competitive enzyme immunoassay^37^ and no immunoreactivity was found in PrRP‐deficient mice.^35^

### 2.9 Statistical analysis

Statistical analyses were performed using Prism, version 9 (GraphPad Software Inc., San Diego, CA, USA) or SPSS (IBM Corp., Armonk, MY, USA). Data are expressed as the mean ± SEM. Number of c‐Fos‐positive cells, increase in water intake, increase in body weight and plasma FGF21 concentrations after AAV injections, which were found to have a non‐normal distribution, were analysed by the Kruskal‐Wallis test followed by post‐hoc Dunn's test or the Mann‐Whitney *U* test. Number of c‐Fos‐positive cells after social defeat stress and plasma concentrations of FGF21 for FGF21‐deficient mice were analysed by the Mann‐Whitney *U* test. Other data were analysed by one‐way factorial ANOVA, two‐way factorial ANOVA followed by post‐hoc Bonferroni's test or repeated measures two‐way ANOVA followed by post‐hoc Bonferroni's test. *P* < 0.05 was considered statistically significant.

## 3 RESULTS

### 3.1 Effects of i.c.v. administration of FGF21 on expression of c‐Fos protein in tyrosine hydroxylase‐positive cells of the nucleus tractus solitarius and area postrema

Effects of i.c.v. administration of FGF21 on activation of catecholaminergic neurones in the medulla oblongata were investigated in rats by examining the expression of c‐Fos protein in tyrosine hydroxylase‐positive cells in the nucleus tractus solitarius and area postrema.

**FIGURE 1:**
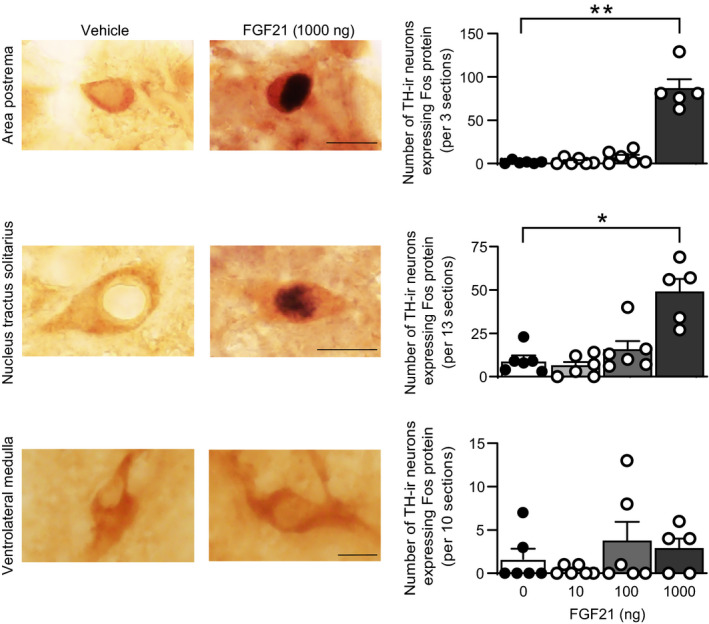
Effects of i.c.v. administration of FGF21 on expression of c‐Fos protein in tyrosine hydroxylase‐positive cells in the nucleus tractus solitarius and area postrema. Photographs of sections containing cells expressing c‐Fos protein‐positive nuclear profiles (black nuclear profiles) and tyrosine hydroxylase‐immunoreactive (TH‐ir) cytoplasmic profiles (brown profiles) in the nucleus tractus solitarius or area postrema of 1000 ng FGF21‐injected and vehicle‐injected rats (left columns). The number of tyrosine hydroxylase‐positive cells expressing c‐Fos protein in the nucleus tractus solitarius and the number of tyrosine hydroxylase‐positive cells expressing c‐Fos protein in the area postrema are shown (right column). Intracerebroventricular administration of FGF21 at a dose of 1000 ng increased the number of tyrosine hydroxylase‐positive cells expressing c‐Fos protein, suggesting that FGF21 activated catecholaminergic cells in the medulla oblongata. Error bars indicate the SEM. The number of animals injected with 0, 10, 100 and 1000 ng FGF21 was 6, 6, 6 and 5, respectively. Scale bars = 10 µm. *P < 0.05, **P < 0.01 vs control animals. Kruskal‐Wallis test followed by post‐hoc Dunn's test

Intracerebroventricular administration of FGF21 at a dose of 1000 ng increased the number of tyrosine hydroxylase‐positive cells expressing c‐Fos protein in the nucleus tractus solitarius (*P* = 0.0063, Kruskal‐Wallis test; 1000 ng vs vehicle, *P* = 0.017, post‐hoc Dunn's multiple comparison test) and in the area postrema (*P* = 0.004, Kruskal‐Wallis test; 1000 ng vs vehicle, *P* = 0.0044, post‐hoc Dunn's multiple comparison test) (Figure 1). Approximately one‐third of noradrenergic neurones co‐express PrRP.^38^, ^39^ We also examined the expression of c‐Fos protein in PrRP‐positive cells in the nucleus tractus solitarius. Administration of FGF21 did not significantly affect the number of PrRP‐positive cells expressing c‐Fos protein (*P* = 0.681, Kruskal‐Wallis test) (Table 1). No significant change was also found in the number of tyrosine hydroxylase‐positive neurones expressing c‐Fos protein in the ventrolateral medulla, where no expression of β‐Klotho has been reported (*P* = 0.566, Kruskal‐Wallis test). These findings suggest that FGF21 activates non‐PrRP catecholaminergic cells in the dorsal part of the caudal medulla oblongata.

**TABLE 1:** Effects of i.c.v. administration of FGF21 on expression of c‐Fos protein in prolactin‐releasing peptide (PrRP)‐positive cells in the nucleus tractus solitarius

*FGF21*	0 ng	10 ng	100 ng	1000 ng
c‐Fos‐positive PrRP cells	3.3 ± 2.1	4.5 ± 1.8	2.5 ± 0.8	1.6 ± 1.0

Note

The number of PrRP‐positive cells expressing c‐Fos protein per animal (7 sections) is shown as the mean ± SEM.

### 3.2 Single in situ hybridisation and double in situ hybridisation of *tyrosine hydroxylase* mRNA and *β‐Klotho* mRNA in the nucleus tractus solitarius

**FIGURE 2:**
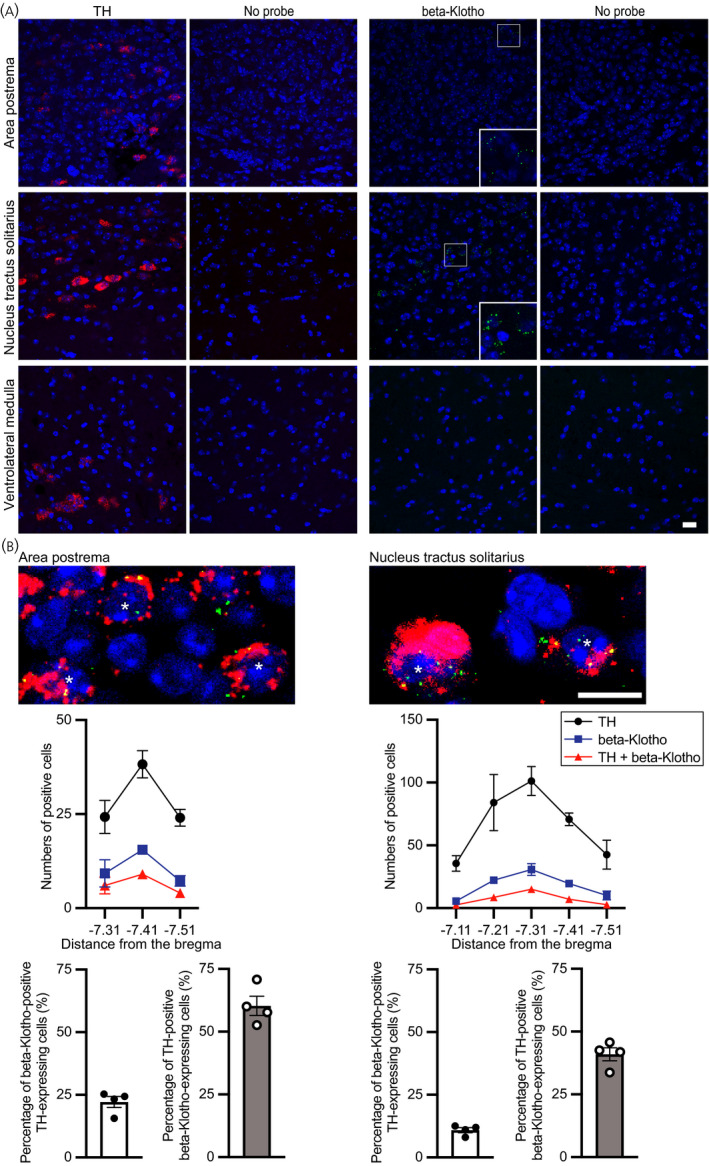
Expression of *β‐Klotho* mRNA in tyrosine hydroxylase (TH) mRNA‐positive cells in the area postrema and nucleus tractus solitarius. A, Single fluorescence in situ hybridisation of *Th* mRNA (red) and *β‐Klotho* mRNA (green) and nuclear counterstaining with NucBlue (blue). Single in situ hybridisation showed *Th* mRNA in the area postrema, nucleus tractus solitarius and ventrolateral medulla (red cytosolic profiles). *β‐Klotho* mRNA was detected in the area postrema and nucleus tractus solitarius (green dots). Magnified photographs for areas in framed boxes were inserted in the lower right corners. B, Double‐labelled fluorescence in situ hybridisation for *Th* (red) and *β‐Klotho* (green) and nuclear counterstaining with NucBlue (blue). Double in situ hybridisation showed that *β‐Klotho* mRNA was expressed in *Th* mRNA‐positive cells. Asterisks show double‐labelled cells. The number of animals for single in situ hybridisation was 2 and the number for double in situ hybridisation was 4. Scale bars = 10 µm

Fluorescence in situ hybridisation was used to determine whether catecholaminergic neurones in the medulla oblongata express *β‐Klotho* mRNA. Expression of *tyrosine hydroxylase* gene was confirmed in the area postrema, nucleus tractus solitarius and ventrolateral medulla. *β‐Klotho* mRNA was expressed in the area postrema and nucleus tractus solitarius but not in the ventrolateral medulla (Figure 2A) as reported previously.^12^ In the area postrema and nucleus tractus solitarius, some β*‐Klotho* mRNA was detected in cells expressing *tyrosine hydroxylase* gene. Cellular co‐expression of *β‐Klotho* and *tyrosine hydroxylase* mRNA was quantitatively evaluated. In the area postrema, 22.1% of tyrosine hydroxylase‐expressing cells also showed expression of *β‐Klotho* mRNA and 60.3% of β‐Klotho‐expressing cells also showed expression of *tyrosine hydroxylase* mRNA. In the nucleus tractus solitarius, 10.9% of tyrosine hydroxylase‐expressing cells also showed expression of *β‐Klotho* mRNA and 41.0% of β‐Klotho‐expressing cells also showed expression of *tyrosine hydroxylase* mRNA (Figure 2B). These findings suggest that significant amounts of β‐Klotho were expressed in catecholaminergic neurones.

### 3.3 Effects of social defeat stress on plasma FGF21 concentrations

**FIGURE 3:**
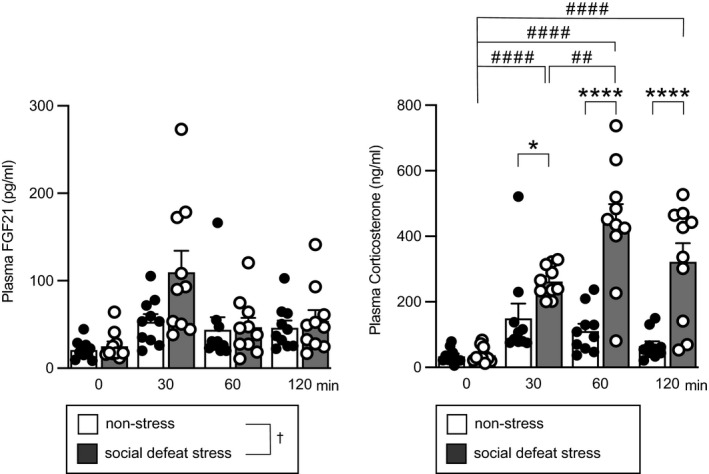
Plasma concentrations of FGF21 and corticosterone after social defeat stress. Plasma FGF21 concentrations after social defeat stress and plasma corticosterone concentrations after social defeat stress are shown. Mice received 10 minutes of social defeat stress once per day. Plasma concentrations 24 hour after the fourth social defeat stress (0) and 30 min (30), 60 min (60) and 120 min (120) after initiation of the fifth social defeat stress were determined. Non‐stress rats were placed in a test cage but received no social defeat stress. Plasma FGF21 concentrations were significantly higher in the social defeat stress group compared to the non‐stress group. Plasma corticosterone concentrations were significantly higher after social defeat stress compared to the concentrations in the non‐stress group and those before social defeat stress. The number of animals in each group was 10. †*P* < 0.05 (left), social defeat stress vs non‐stress, two‐way factorial ANOVA. *P < 0.05, ****P < 0.0001, stress group vs non‐stress group at corresponding time points; ##*P* < 0.01 vs after the fifth stress (30 min), ####*P* < 0.0001 vs before the fifth stress (0 min) in the stress group, two‐way factorial ANOVA followed by Bonferroni's test

Effects of social defeat stress on plasma FGF21 concentrations were examined. Male C57BL/6J mice were exposed to aggressive ICR mice as social defeat stress once per day. Plasma concentrations 24 hours after the fourth social defeat stress (0 minutes) and 30 minutes, 60 minutes and 120 minutes after the fifth social defeat stress were determined. Plasma FGF21 concentrations were significantly higher after social defeat stress than the concentrations in mice before the fifth social stress or those in non‐stressed control mice (significant effect of group *F*_1,72_ = 4.344, *P* = 0.0407; significant effect of time *F*_3,72_ = 7.758, *P* = 0.000147; no significant interaction, *F*_3,72_ = 2.260, *P* = 0.0888; two‐way factorial ANOVA; 30 minutes vs 0 minutes, *P* = 3.35 × 10^−5^, Bonferroni's test) (Figure 3, left), suggesting that social defeat stress increases FGF21 in plasma.

Plasma concentrations of corticosterone were also increased after social defeat stress (significant effect of group *F*_1,72_ = 52.030, *P* = 4.46 × 10^−10^; significant effect of time *F*_3,72_ = 17.34, *P* = 1.42 × 10^−8^; significant interaction, *F*_3,72_ = 8.928, *P* = 4.19 × 10^−5^; two‐way factorial ANOVA; 30 minutes in social defeat group vs 0 minute in social defeat group, *P* = 9.59 × 10^−5^; 60 minutes in social defeat group vs 0 minute in social defeat group, *P* = 3.05 × 10^−11^; 120 minutes in social defeat group vs 0 minute in social defeat group, *P* = 7.57 × 10^−7^; 60 minutes in social defeat group vs 30 minutes in social defeat group, *P* = 3.17 x 10^‐3^; 30 minutes in social defeat group vs 30 minutes in non‐stress group, *P* = 0.0234; 60 minutes in social defeat group vs 60 minutes in non‐stress group, *P* = 3.32 × 10^−9^; 120 minutes in social defeat group vs 120 minutes in non‐stress group, *P* = 1.25 × 10^−6^, Bonferroni's test) (Figure 3, right).

### 3.4 Effects of social defeat stress on expression of c‐Fos protein in tyrosine hydroxylase‐positive cells of the nucleus tractus solitarius and area postrema

Effects of social defeat stress on activation of catecholaminergic neurones in the medulla oblongata were investigated in mice by examining the expression of c‐Fos protein in tyrosine hydroxylase‐positive cells in the nucleus tractus solitarius and area postrema.

**FIGURE 4:**
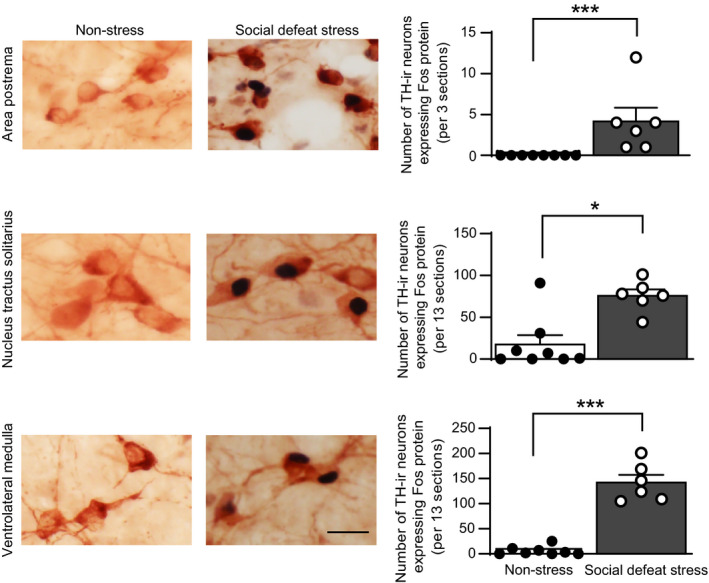
Effects of social defeat stress on expression of c‐Fos protein in tyrosine hydroxylase‐positive cells in the area postrema, nucleus tractus solitarius and ventrolateral medulla. Photographs of sections containing cells expressing c‐Fos protein‐positive nuclear profiles and tyrosine hydroxylase‐immunoreactive (TH‐ir) cytoplasmic profiles in the area postrema, nucleus tractus solitarius and ventrolateral medulla in control mice and in mice after social defeat stress are shown (left). The number of tyrosine hydroxylase‐positive cells expressing c‐Fos protein in the area postrema, nucleus tractus solitarius and ventrolateral medulla is shown (right column). Social defeat stress increased the number of tyrosine hydroxylase‐positive cells expressing c‐Fos protein, suggesting that social defeat stress activated catecholaminergic cells in the medulla oblongata. Error bars indicate the SEM. The number of control mice and mice that received social defeat stress was 8 and 6, respectively. *P < 0.05, ***P < 0.001 vs control animals. Mann Whitney *U* test. Scale bar = 25 µm

In mice after social defeat stress, the number of tyrosine hydroxylase‐positive neurones expressing c‐Fos protein in the area postrema (*U* = 0, *P* = 0.0003, Mann Whitney *U* test), nucleus tractus solitarius (*U* = 5, *P* = 0.0127, Mann Whitney *U* test) and ventrolateral medulla (*U* = 0, *P* = 0.0007, Mann Whitney *U* test) was significantly greater compared to non‐stressed mice (Figure 4), suggesting that social defeat stress activates catecholaminergic neurones in the dorsal and ventral parts of the caudal medulla oblongata.

### 3.5 Effects of FGF21 deficiency on water intake, food intake, body weight and behaviours after social defeat stress

The roles of endogenous FGF21 in the control of water intake, body weight, food intake and anxiety‐related or depression‐like behaviour in response to social defeat stress were then examined using FGF21‐deficient mice. These parameters were assessed before and after application of social defeat stress.

**FIGURE 5:**
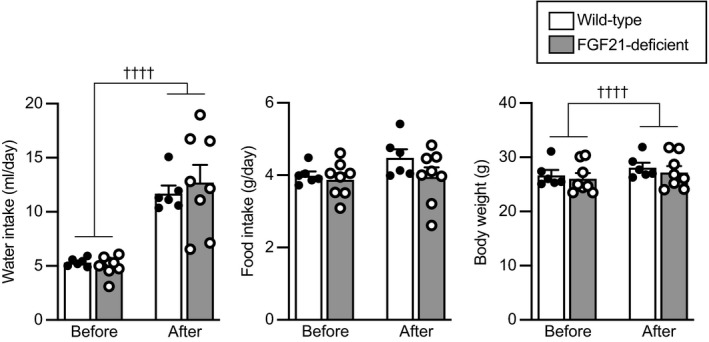
Amount of water intake, amount of food intake and body weight before and after social defeat stress in wild‐type mice and FGF21‐deficient mice. Amount of water intake per day and body weight were increased after social defeat stress compared to before social defeat stress. No significant difference was found between wild‐type mice and FGF21‐deficient mice. The number of wild‐type mice and FGF21‐deficient mice was 6 and 8, respectively. ††††*P* < 0.0001, before stress vs after stress, two‐way repeated measures ANOVA

Water intake was recorded because FGF21 has been shown to be involved in water intake. Administration of FGF21 has been reported to increase water intake^40^ and FGF21 deficiency blocked metabolic stress‐induced water intake.^41^ Water intake was increased after social defeat stress compared to before social defeat stress, and no significant difference was found between wild‐type and FGF21‐deficient mice (no significant effect of group, *F*_1,12_ = 0.101, *P* = 0.756; significant effect of time, *F*_1,12_ = 56.968, *P* = 6.79 × 10^−6^; no significant interaction, *F*_1,12_ = 0.533, *P* = 0.479; repeated measures two‐way ANOVA) (Figure 5, left).

Food intake was not significantly different after social defeat stress compared to before social defeat stress, and no significant difference was found between wild‐type and FGF21‐deficient mice (no significant effect of group, *F*_1,12_ = 1.809, *P* = 0.203; no significant effect of time *F*_1,12_ = 2.714, *P* = 0.125; no significant interaction, *F*_1,12_ = 1.444, *P* = 0.253; repeated measures two‐way ANOVA) (Figure 5, middle).

Body weight was significantly increased after social defeat stress compared to before social defeat stress, and no significant difference was found between wild‐type and FGF21‐deficient mice (no significant effect of group *F*_1,12_ = 0.273, *P* = 0.611; significant effect of time *F*_1,12_ = 50.943, *P* = 1.18 × 10^−5^; no significant interaction, *F*_1,12_ = 0.395, *P* = 0.542; repeated measures two‐way ANOVA) (Figure 5, right).

#### 3.5.1 Behaviours during exposure to aggressor mice

Wild‐type or FGF21‐deficient mice were exposed to aggressive ICR mice for 10 minutes per day for 5 days. Durations of aggressive behaviours received, defeat posture, escape behaviour and immobility behaviour were measured during the first and fifth sessions of social defeat stress.

**FIGURE 6:**
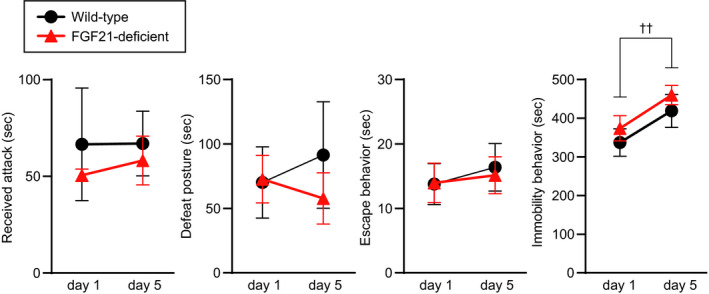
Duration of attacks received, social defeat posture, escape behaviour and immobility behaviour during exposure to aggressor ICR mice. No significant difference was found in durations for attacks received, defeat posture, immobility behaviour and escape behaviour between wild‐type mice and FGF21‐deficient mice. The time spent for immobility behaviour was increased during the fifth social defeat stress compared to during the first social defeat stress. The number of wild‐type mice and FGF21‐deficient mice was 6 and 8, respectively. ††*P* < 0.01, day 1 vs day 5, two‐way repeated measures ANOVA

The duration of attacks received during exposure to aggressors was not significantly different between wild‐type and FGF21‐deficient mice (no significant effect of group *F*_1,12_ = 0.724, *P* = 0.411; no significant effect of time *F*_1,12_ = 0.052, *P* = 0.823; no significant interaction, *F*_1,12_ = 0.041, *P* = 0.843; repeated measures two‐way ANOVA) (Figure 6). Durations of defeat posture, escape behaviour and immobility behaviour were also not significantly different between wild‐type and FGF21‐deficient mice (defeat posture, no significant effect of group *F*_1,12_ = 0.212, *P* = 0.654; no significant effect of time *F*_1,12_ = 0.039, *P* = 0.847; no significant interaction, *F*_1,12_ = 1.281, *P* = 0.280; escape behaviour, no significant effect of group *F*_1,12_ = 0.016, *P* = 0.901; no significant effect of time *F*_1,12_ = 1.129, *P* = 0.309; no significant interaction, *F*_1,12_ = 0.157, *P* = 0.699; immobility behaviour, no significant effect of group *F*_1,12_ = 0.936, *P* = 0.352; significant effect of time *F*_1,12_ = 10.823, *P* = 0.00646; no significant interaction, *F*_1,12_ = 0.00617, *P* = 0.939; repeated measures two‐way ANOVA).

#### 3.5.2 Elevated plus maze test

Anxiety‐related behaviour was assessed by an elevated plus maze test.

**FIGURE 7:**
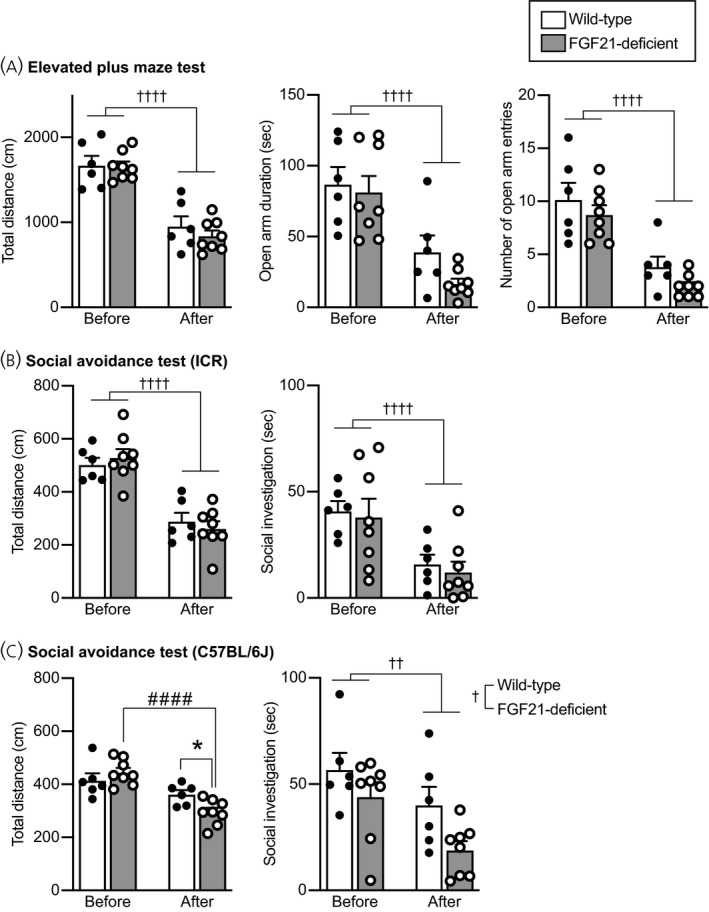
Anxiety‐related behaviours in an elevated plus maze test and social avoidance behaviours in wild‐type and FGF21‐deficient mice. A, Total distance of locomotion (Total distance), time spent for staying in the open arms (Open arm duration), and number of entries into the open arms (Number of open arm entries) in an elevated plus maze test. The values of these parameters were decreased after social defeat stress compared to the values before social defeat stress. No significant difference was found between wild‐type mice and FGF21‐deficient mice. B, Total distance of locomotion and time spent for social contact with ICR mice in a social avoidance test. Total locomotor distance and time spent for investigating a cylinder containing an ICR mouse were reduced after social defeat stress, and no significant difference was found between wild‐type mice and FGF21‐deficent mice. C, Total distance of locomotion and time spent for social contact with a C57BL/6J mouse in a social avoidance test. Total locomotion activity was reduced after social defeat stress in FGF21‐deficient mice but not in wild‐type mice, and locomotor activity after stress was significantly lower in FGF21‐deficient mice compared to wild‐type mice. The time spent for investigating a cylinder containing a C57BL/6J mouse was significantly reduced after social defeat stress, and it was significantly shorter in FGF21‐deficient mice compared to wild‐type mice. The number of wild‐type mice and FGF21‐deficient mice was 6 and 8, respectively. ††*P* < 0.01, ††††*P* < 0.0001, before stress vs after stress; †*P* < 0.05, wild‐type mice vs FGF21‐deficient mice, two‐way repeated measures ANOVA. #### *P* < 0.0001, FGF21‐deficient mice before stress vs FGF21‐deficient mice after stress; *P < 0.05, wild‐type mice after stress vs FGF21‐deficient mice after stress, two‐way repeated measures ANOVA followed by post‐hoc Bonferroni's test

Locomotor activity during an elevated plus maze test was significantly reduced after social defeat stress compared to before social defeat stress, and no significant difference was found between wild‐type and FGF21‐deficient mice (no significant effect of group *F*_1,12_ = 0.855, *P* = 0.373; significant effect of time *F*_1,12_ = 60.098, *P* = 5.18 × 10^−6^; no significant interaction, *F*_1,12_ = 0.261, *P* = 0.619; repeated measures two‐way ANOVA) (Figure 7A, left).

Time spent staying in open arms was significantly reduced after social defeat stress compared to before social defeat stress, and no significant difference was found between wild‐type and FGF21‐deficient mice (no significant effect of group *F*_1,12_ = 1.889, *P* = 0.194; significant effect of time *F*_1,12_ = 33.068, *P* = 9.16 × 10^−5^; no significant interaction, *F*_1,12_ = 0.737, *P* = 0.407; repeated measures two‐way ANOVA) (Figure 7A, middle).

The number of entries into open arms was significantly reduced after social defeat stress compared to before social defeat stress, and no significant difference was found between wild‐type and FGF21‐deficient mice (no significant effect of group *F*_1,12_ = 2.907, *P* = 0.114; significant effect of time *F*_1,12_ = 44.905, *P* = 2.19 × 10^−5^; no significant interaction, *F*_1,12_ = 0.0455, *P* = 0.835; repeated measures two‐way ANOVA) (Figure 7A, right).

These findings suggest that social defeat stress increased anxiety‐related behaviours and that FGF21 is not indispensable for increased anxiety‐related behaviour observed after social defeat stress.

#### 3.5.3 Social avoidance test

Social avoidance from mice of the ICR strain, comprising the strain of mice from which the test mice received attacks, was assessed by examining time spent for social investigation towards a cylinder containing a novel ICR male mouse. Social avoidance from mice of the C57BL/6J strain, the same strain as that of the test mice, was assessed by examining time spent for social investigation towards a cylinder containing a novel C57BL/6J male mouse. The cumulative duration that test mice spent for sniffing towards perforated parts of the cylinders was determined.

Locomotor activity during a social avoidance test with an ICR mouse was significantly reduced after social defeat stress compared to before social defeat stress, and no significant difference was found between wild‐type and FGF21‐deficient mice (no significant effect of group *F*_1,12_ = 0.000119, *P* = 0.991; significant effect of time *F*_1,12_ = 105.685, *P* = 2.65 × 10^−7^; no significant interaction, *F*_1,12_ = 1.334, *P* = 0.271; repeated measures two‐way ANOVA) (Figure 7B, left).

Investigation time towards the cylinder containing an ICR mouse was significantly reduced after social defeat stress compared to before social defeat stress, and no significant difference was found between wild‐type and FGF21‐deficient mice (no significant effect of group *F*_1,12_ = 0.154, *P* = 0.702; significant effect of time *F*_1,12_ = 58.097, *P* = 6.15 × 10^−6^; no significant interaction, *F*_1,12_ = 0.0171, *P* = 0.898; repeated measures two‐way ANOVA) (Figure 7B, right).

Locomotor activity during a social avoidance test with a C57BL/6J mouse after social defeat stress compared to before social defeat stress was not significantly reduced in wild‐type mice but was reduced in FGF21‐deficient mice, and it was significantly smaller in FGF21‐deficient mice compared to wild‐type mice (no significant effect of group *F*_1,12_ = 0.911, *P* = 0.359; significant effect of time *F*_1,12_ = 28.164, *P* = 0.000186; significant interaction, *F*_1,12_ = 6.640, *P* = 0.0242; repeated measures two‐way ANOVA; before stress in wild‐type mice vs after stress in wild‐type mice, *P* = 0.0961; before stress in FGF21‐deficient mice vs after stress in FGF21‐deficient mice, *P* = 6.02 × 10^−5^; before stress in wild‐type mice vs before stress in FGF21‐deficient mice, *P* = 0.335; after stress in wild‐type mice vs after stress in FGF21‐deficient mice, *P* = 0.0148; post‐hoc Bonferroni's multiple comparison test) (Figure 7C, left).

Investigation time towards the cylinder containing a C57BL/6J mouse was significantly reduced after social defeat stress compared to before social defeat stress, and it was significantly reduced in FGF21‐deficient mice compared to wild‐type mice (significant effect of group *F*_1,12_ = 4.889, *P* = 0.0472; significant effect of time *F*_1,12_ = 13.012, *P* = 0.00360; no significant interaction, *F*_1,12_ = 0.539, *P* = 0.477; repeated measures two‐way ANOVA) (Figure 7C, right).

The findings suggest that social defeat stress induced social avoidance from ICR mice in both wild‐type and FGF21‐deficient mice and that avoidance from C57BL/6J mice appeared to be greater in FGF21‐deficient mice compared to wild‐type‐mice, being consistent with the idea that FGF21 attenuates social defeat stress‐induced social avoidance.

#### 3.5.4 Social interaction test

**FIGURE 8:**
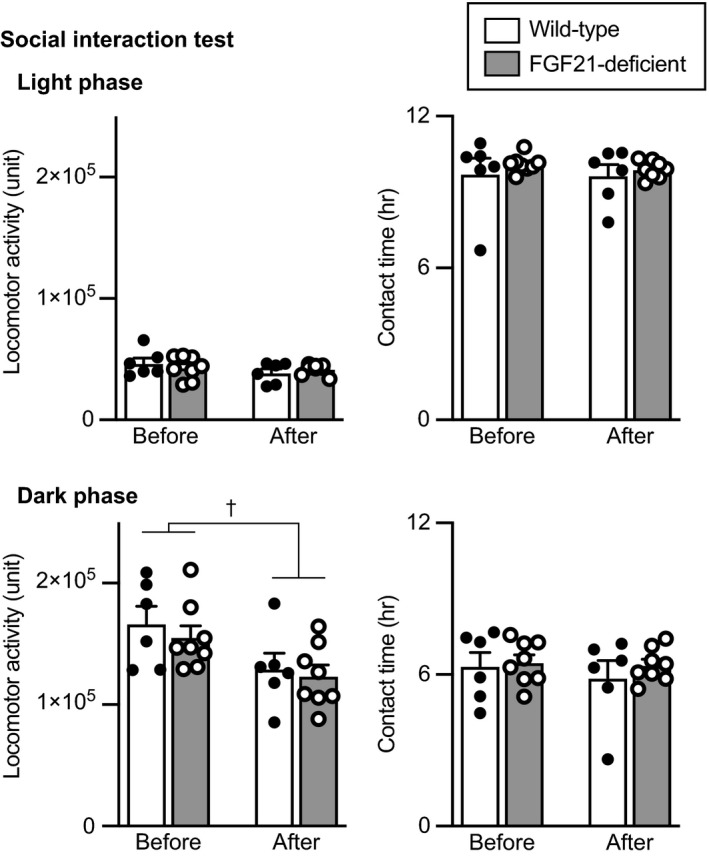
Locomotor activity and time spent in contact in a social interaction test. Locomotor activity and time spent staying in contact with each other during the light phase (upper) and during the dark phase (lower) in wild‐type mice and FGF21‐deficient mice are shown. Locomotor activity during the dark phase was reduced after social defeat stress compared to that before social defeat stress. No significant difference was found between wild‐type mice and FGF21‐deficent mice. The number of wild‐type mice and FGF21‐deficient mice was 6 and 8, respectively. †*P* < 0.05 before stress vs after stress, two‐way repeated measures ANOVA

Social contact in a familiar environment was assessed in a social interaction test. Locomotor activity and duration of social contact in test cages were measured during the period when mice were kept in pairs with native male C57BL/6J mice. During the light phase, locomotor activity and time spent in contact with each other were not significantly changed after social defeat stress, and no significant difference was found between wild‐type mice and FGF21‐deficient mice (locomotor activity during the light phase, no significant effect of group, *F*_1,12_ = 0.00627, *P* = 0.938; no significant effect of time (before vs after), *F*_1,12_ = 2.620, *P* = 0.131; no significant interaction, *F*_1,12_ = 1.492, *P* = 0.245; contact time during the light phase, no significant effect of group *F*_1,12_ = 0.546, *P* = 0.474; no significant effect of time *F*_1,12_ = 0.510, *P* = 0.489; no significant interaction, *F*_1,12_ = 0.115, *P* = 0.740; repeated measures two‐way ANOVA) (Figure 8, upper).

During the dark phase, locomotor activity was reduced after social defeat stress and no significant difference was found between wild‐type and FGF21‐deficient mice (no significant effect of group *F*_1,12_ = 0.751, *P* = 0.403; significant effect of time *F*_1,12_ = 7.506, *P* = 0.0179; no significant interaction, *F*_1,12_ = 0.049, *P* = 0.828; repeated measures two‐way ANOVA) (Figure 8, lower). The time spent in contact with each other during the dark phase was not significantly changed after social defeat stress and no significant difference was found between wild‐type and FGF21‐deficient mice (no significant effect of group, *F*_1,12_ = 0.364, *P* = 0.557; no significant effect of time, *F*_1,12_ = 0.933, *P* = 0.353; no significant interaction, *F*_1,12_ = 0.362, *P* = 0.559; repeated measures two‐way ANOVA) (Figure 8, lower right).

The findings suggest that social defeat stress decreased locomotor activity during the dark phase but did not significantly affect contact time with C57BL/6J mice both in wild‐type and FGF21‐deficient mice.

#### 3.5.5 Forced swimming test

Depression‐like behaviour was assessed by a forced swimming test.

**FIGURE 9:**
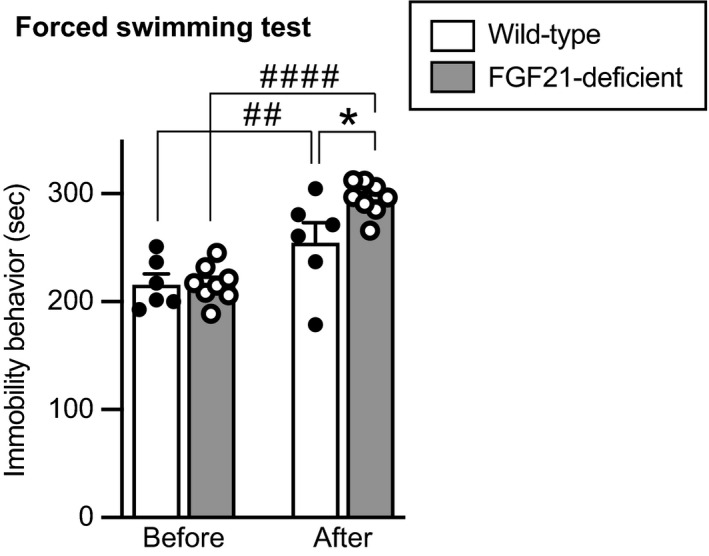
Duration of immobility behaviour of wild‐type mice and FGF21‐deficient mice in a forced swimming test. The time spent for immobility behaviour in a forced swimming test was increased after social defeat stress compared to that before social defeat stress. Immobility behaviour after social defeat stress was augmented in FGF21‐defecient mice compared to that in wild‐type mice. The number of wild‐type mice and FGF21‐deficient mice was 6 and 8, respectively. *P < 0.05, wild‐type mice after stress vs FGF21‐deficient mice after stress; ## *P* < 0.01, wild‐type mice before stress vs wild‐type mice after stress; ####*P* < 0.0001, FGF21‐deficient mice before stress vs FGF21‐deficient mice after stress, two‐way repeated measures ANOVA followed by Bonferroni's test

The time spent for immobility behaviour in a forced swimming test was increased after social defeat stress, and the time spent for immobility behaviour after social defeat stress was significantly longer in FGF21‐deficient mice compared to wild‐type mice (no significant effect of group, *F*_1,12_ = 2.796, *P* = 0.120; significant effect of time, *F*_1,12_ = 73.687, *P* = 1.81 × 10^−6^; significant interaction, *F*_1,12_ = 8.520, *P* = 0.0129; repeated measures two‐way ANOVA; before stress in wild‐type mice vs after stress in wild‐type mice, *P* = 0.00278; before stress in FGF21‐deficient mice vs after stress in FGF21‐deficient mice, *P* = 1.42 × 10^−6^; before stress in wild‐type mice vs before stress in FGF21‐deficient mice, *P* = 0.986; after stress in wild‐type mice vs after stress in FGF21‐deficient mice, *P* = 0.0315; post‐hoc Bonferroni's test) (Figure 9), being consistent with the idea that FGF21 has anti‐depressant actions.

### 3.6 Effects of FGF21 overexpression on water intake, food intake, body weight and behaviours after social defeat stress in wild‐type mice

Effects of exogenous FGF21 were examined by use of an intraperitoneal injection of AAV vectors.

**FIGURE 10:**
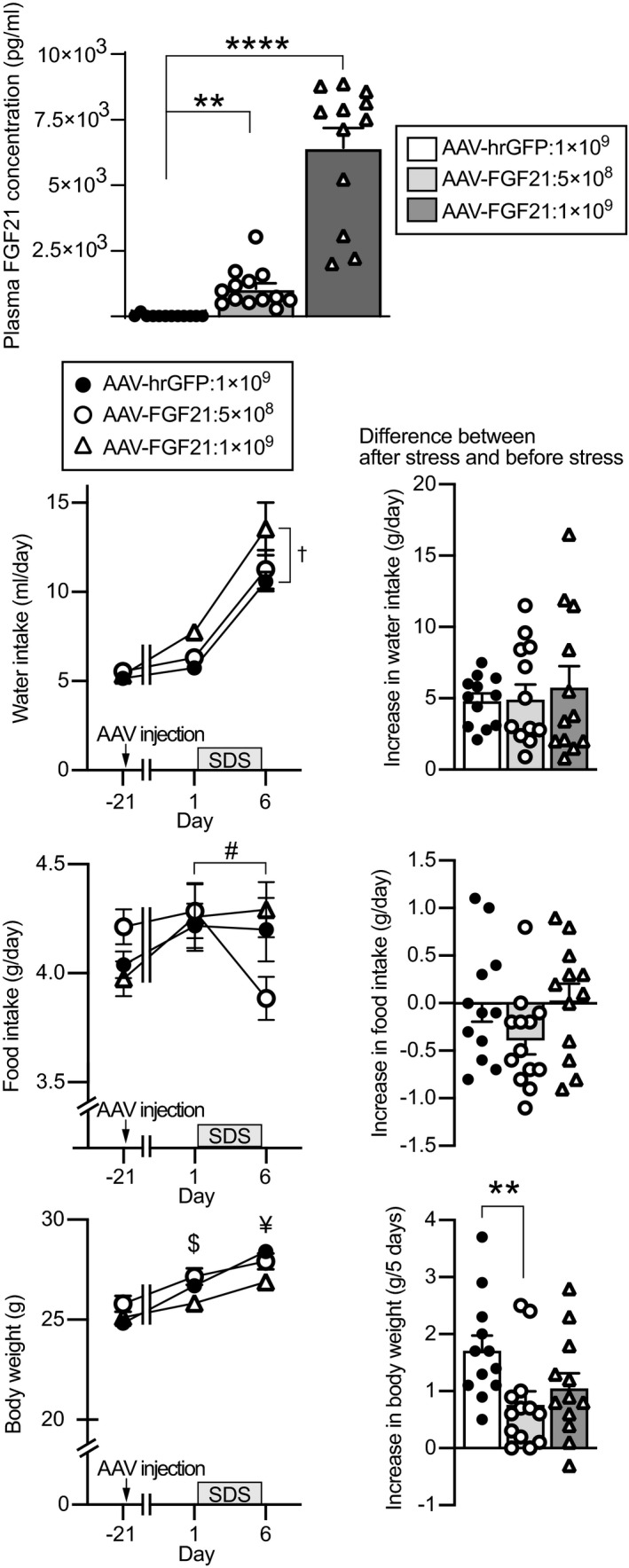
Plasma FGF21 concentrations, water intake, body weight and food intake in mice injected with AAV‐FGF21. Plasma concentrations of FGF21 in mice injected with AAV‐hrGFP, 5 × 10^8^ vg AAV‐FGF21 and 1 × 10^9^ vg AAV‐FGF21 at 33 days after AAV injection are shown (upper). The plasma concentrations of FGF21 were significantly higher in the mice injected with 1 × 10^9^ vg AAV‐FGF21 and in the mice injected with 5 × 10^8^ vg AAV‐FGF21 compared to the mice injected with AAV‐hrGFP. Amount of water intake per day, amount of food intake per day and body weight before AAV injection, before social defeat stress and after social defeat stress (lower left) and increase in water intake per day, increase in food intake per day and increase in body weight during social defeat stress for 5 days (lower right) are shown. Water intake was greater in mice injected with AAV‐FGF21 1 × 10^9^ vg compared to mice injected with AAV‐hrGFP. Body weight after social defeat stress was significantly lower in mice injected with AAV‐FGF21 1 × 10^9^ vg. Increase in body weight during social defeat stress was significantly smaller in mice injected with AAV‐FGF21 5 × 10^8^ vg compared to mice injected with AAV‐hrGFP. The number of mice injected with AAV‐hrGFP, 5 × 10^8^ vg AAV‐FGF21 and 1 × 10^9^ vg AAV‐FGF21 was 12, 13 and 12, respectively. **P < 0.01, ****P < 0.0001 vs AAV‐hrGFP‐injected mice, Kluskal‐Wallis test followed by Dunn's test. †*P* < 0.05, AAV‐FGF21 1 × 10^9^ vg‐injected mice vs AAV‐hrGFP‐injected mice, two‐way repeated measures ANOVA. #*P* < 0.05, before stress in AAV‐FGF21 5 × 10^8^ vg‐injected mice vs after stress in AAV‐FGF21 5 × 10^8^ vg‐injected mice; $*P* < 0.05, before stress in AAV‐FGF21 1 × 10^9^ vg‐injected mice vs before stress in AAV‐FGF21 5 × 10^8^ vg‐injected mice; ¥*P* < 0.05, after stress in AAV‐FGF21 1 × 10^9^ vg‐injected mice vs after stress in AAV‐hrGFP‐injected mice; two‐way repeated measures ANOVA followed by Bonferroni's test. Absolute values of water intake, food intake and body weight were analysed by two‐way repeated measures ANOVA followed by Bonferroni's test. Plasma FGF21 concentrations and increases in water intake and body weight after stress were analysed by Kluskal‐Wallis test followed by Dunn's test. Increase in food intake was analysed by one‐way factorial ANOVA. SDS, social defeat stress

Plasma FGF21 concentrations in mice that had been injected with AAV‐hrGFP at 1 × 10^9^ vg per 500 μL and with AAV‐FGF21 at 5 × 10^8^ vg and 1 × 10^9^ vg per 500 μL were 34.7, 1059.31 and 6437.87 pg mL^‐1^, respectively (*P* < 0.0001; Kruskal–Wallis test; AAV‐hrGFP vs AAV‐FGF21 5 × 10^8^, *P* = 0.0064; AAV‐hrGFP vs AAV‐FGF21 1 × 10^9^ vg, *P* < 0.0001, post‐hoc Dunn's test) (Figure 10, first row).

Water intake was increased in mice injected with 1 × 10^9^ vg compared to mice injected with AAV‐hrGFP and it was also increased after social defeat stress (significant effect of group, *F*_2,34_ = 4.599, *P* = 0.0171; significant effect of time, *F*_1.09,37.20_ = 84.659, *P* = 1.50 × 10^−11^; no significant interaction, *F*_2.19,37.20_ = 1.438, *P* = 0.250; repeated measures two‐way ANOVA; AAV‐hrGFP vs 1 × 10^9^ vg AAV‐FGF21, *P* = 0.0165; before injection vs before stress, *P* = 1.068 × 10^−8^; before stress vs after stress, *P* = 2.78 × 10^−9^; post‐hoc Bonferroni's test) (Figure 10, second row). No significant difference was found in increase in water intake after social defeat stress among the groups (*P* = 0.879; Kruskal‐Wallis test) (Figure 10, second row right).

Food intake was not significantly different among mice injected with AAV‐hrGFP or AAV‐FGF21 (no significant effect of group, *F*_2,34_ = 0.103, *P* = 0.902; no significant effect of time, *F*_2,68_ = 2.363, *P* = 0.102; significant interaction, *F*_4,68_ = 2.899, *P* = 0.0282; repeated measures two‐way ANOVA, no significant difference among groups at the time points of before AAV, before stress and after stress; before stress in AAV‐FGF21 5 × 10^8^ vs after stress in AAV‐FGF21 5 × 10^8^, *P* = 0.049, post‐hoc Bonferroni's test) (Figure 10, third row left). No significant difference was found in food intake changes after social defeat stress among the groups (*F*_2,34_ = 2.187, *P* = 0.128; one‐way factorial ANOVA) (Figure 10, third row right).

Body weight was significantly increased after AAV injection (no significant effect of group *F*_2,34_ = 2.978, *P* = 0.064; significant effect of time *F*_2,68_ = 144.883, *P* = 3.041 × 10^−25^, significant interaction, *F*_4,68_ = 7.587, *P* = 4.091 × 10^−5^; repeated measures two‐way ANOVA; no significant difference among the groups before AAV; before stress in mice injected with AAV‐FGF21 5 × 10^8^ vg vs before stress in mice injected with AAV‐FGF21 1 × 10^9^ vg, *P* = 0.013; after stress in mice injected with AAV‐FGF21 1 × 10^9^ vg vs after stress in mice injected with AAV‐hrGFP, *P* = 0.016; post‐hoc Bonferroni's test). Body weight increase during the 5‐day social defeat stress was significantly smaller in mice injected with AAV‐FGF21 at 5 × 10^8^ vg compared to mice injected with AAV‐hrGFP (*P* = 0.0169, Kruskal‐Wallis test; AAV‐FGF21 1 × 10^9^ vg vs AAV‐hrGFP, *P* = 0.155; AAV‐FGF21 5 × 10^8^ vg vs AAV‐hrGFP, *P* = 0.0093; post‐hoc Dunn's test) (Figure 10, bottom right).

#### 3.6.1 Elevated plus maze test

**FIGURE 11:**
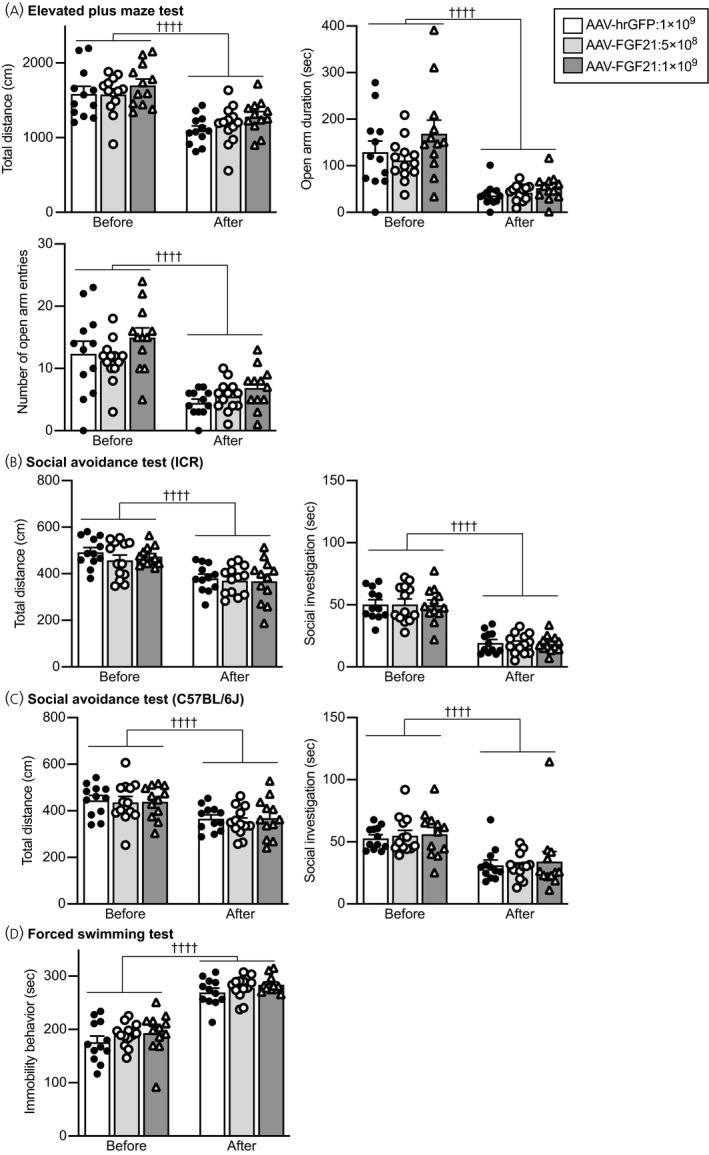
Anxiety‐related behaviours in an elevated plus maze test and social avoidance behaviours and immobility behaviours in a forced swimming test in mice injected with AAV‐hrGFP or AAV‐FGF21. A, Total distance of locomotion (Total distance), time spent for staying in the open arms (Open arm duration) and number of entries into the open arms (Number of open arm entries) in an elevated plus maze test. The values of these parameters were decreased after social defeat stress compared to the values before social defeat stress. No significant difference was found among mice injected with AAV‐hrGFP and mice injected with AAV‐FGF21. B, Total distance of locomotion and time spent for social contact with ICR mice in a social avoidance test. Total locomotor distance and time spent for investigating a cylinder containing an ICR mouse were reduced after social defeat stress, and no significant difference was found among mice injected with AAV‐hrGFP and mice injected with AAV‐FGF21. C, Total distance of locomotion and time spent for social contact with a C57BL/6J mouse in a social avoidance test. Total locomotor distance and time spent for investigating a cylinder containing a C57BL/6J mouse were reduced after social defeat stress, and no significant difference was found among mice injected with AAV‐hrGFP and mice injected with AAV‐FGF21. The number of mice injected with AAV‐hrGFP, 5 × 10^8^ vg AAV‐FGF21 and 1 × 10^9^ vg AAV‐FGF21 was 12, 13 and 12, respectively. The number of wild‐type mice and FGF21‐deficient mice was 6 and 8, respectively. ††††*P* < 0.0001, before stress vs after stress, two‐way repeated measures ANOVA

Locomotor activity during an elevated plus maze test was significantly reduced after social defeat stress compared to before social defeat stress, and no significant difference was found among mice injected with AAV‐hrGFP and mice injected with AAV‐FGF21 (no significant effect of group *F*_2,34_ = 1.590, *P* = 0.219; significant effect of time *F*_1,34_ = 81.342, *P* = 1.53 × 10^−10^; no significant interaction, *F*_2,34_ = 0.253, *P* = 0.778; repeated measures two‐way ANOVA) (Figure 11A).

The time spent staying in open arms was significantly reduced after social defeat stress compared to before social defeat stress, and no significant difference was found among mice injected with AAV‐hrGFP and mice injected with AAV‐FGF21 (no significant effect of group *F*_2,34_ = 2.070, *P* = 0.142; significant effect of time *F*_1,34_ = 61.024, *P* = 4.31 × 10^−9^; no significant interaction, *F*_2,34_ = 1.334, *P* = 0.277; repeated measures two‐way ANOVA).

The number of entries into open arms was significantly reduced after social defeat stress compared to before social defeat stress, and no significant difference was found among mice injected with AAV‐hrGFP and mice injected with AAV‐FGF21 (no significant effect of group *F*_2,34_ = 2.254, *P* = 0.120; significant effect of time *F*_1,34_ = 77.806, *P* = 2.62 × 10^−10^; no significant interaction, *F*_2,34_ = 0.842, *P* = 0.439; repeated measures two‐way ANOVA).

These findings suggest that overexpression of FGF21 did not affect anxiety‐related behaviour observed after social defeat stress.

#### 3.6.2 Social avoidance test

Locomotor activity during a social avoidance test with an ICR mouse was significantly reduced after social defeat stress compared to before social defeat stress, and no significant difference was found among mice injected with AAV‐hrGFP and mice injected with AAV‐FGF21 (no significant effect of group *F*_2,34_ = 0.551, *P* = 0.582; significant effect of time *F*_1,34_ = 52.303, *P* = 2.27 × 10^−8^; no significant interaction, *F*_2,34_ = 0.303, *P* = 0.740; repeated measures two‐way ANOVA) (Figure 11B). Investigation time towards the cylinder containing an ICR mouse was significantly reduced after social defeat stress compared to before social defeat stress, and no significant difference was found among mice injected with AAV‐hrGFP and mice injected with AAV‐FGF21 (no significant effect of group *F*_2,34_ = 0.025, *P* = 0.975; significant effect of time *F*_1,34_ = 165.798, *P* = 1.25 × 10^−14^; no significant interaction, *F*_2,34_ = 0.017, *P* = 0.983; repeated measures two‐way ANOVA).

Locomotor activity during a social avoidance test with a C57BL/6J mouse was significantly reduced after social defeat stress compared to before social defeat stress, and no significant difference was found among mice injected with AAV‐hrGFP and mice injected with AAV‐FGF21 (no significant effect of group *F*_2,34_ = 0.145, *P* = 0.865; significant effect of time *F*_1,34_ = 35.555, *P* = 9.62 × 10^−7^; no significant interaction, *F*_2,34_ = 0.118, *P* = 0.889; repeated measures two‐way ANOVA) (Figure 11C).

Investigation time towards the cylinder containing a C57BL/6J mouse was significantly reduced after social defeat stress compared to before social defeat stress, and no significant difference was found among mice injected with AAV‐hrGFP and mice injected with AAV‐FGF21 (no significant effect of group *F*_2,34_ = 0.211, *P* = 0.811; significant effect of time *F*_1,34_ = 45.352, *P* = 9.74 × 10^−8^; no significant interaction, *F*_2,34_ = 0.101, *P* = 0.904; repeated measures two‐way ANOVA).

The findings suggest that overexpression of FGF21 did not significantly change social avoidance.

#### 3.6.3 Forced swimming test

The time spent for immobility behaviour in a forced swimming test was increased after social defeat stress compared to before social defeat stress, and no significant difference was found among mice injected with AAV‐hrGFP and mice injected with AAV‐FGF21 (no significant effect of group *F*_2,34_ = 1.587, *P* = 0.219; significant effect of time *F*_1,34_ = 264.292, *P* = 1.33 × 10^−17^; no significant interaction, *F*_2,34_ = 0.050, *P* = 0.951; repeated measures two‐way ANOVA) (Figure 11D), suggesting that overexpression of FGF21 did not significantly change immobility behaviour in a forced swimming test.

### 3.7 Effects of FGF21 overexpression on behaviours after social defeat stress in FGF21‐deficient mice

Effects of exogenous FGF21 in FGF21‐deficient mice were examined by use of an intraperitoneal injection of AAV vectors.

**FIGURE 12:**
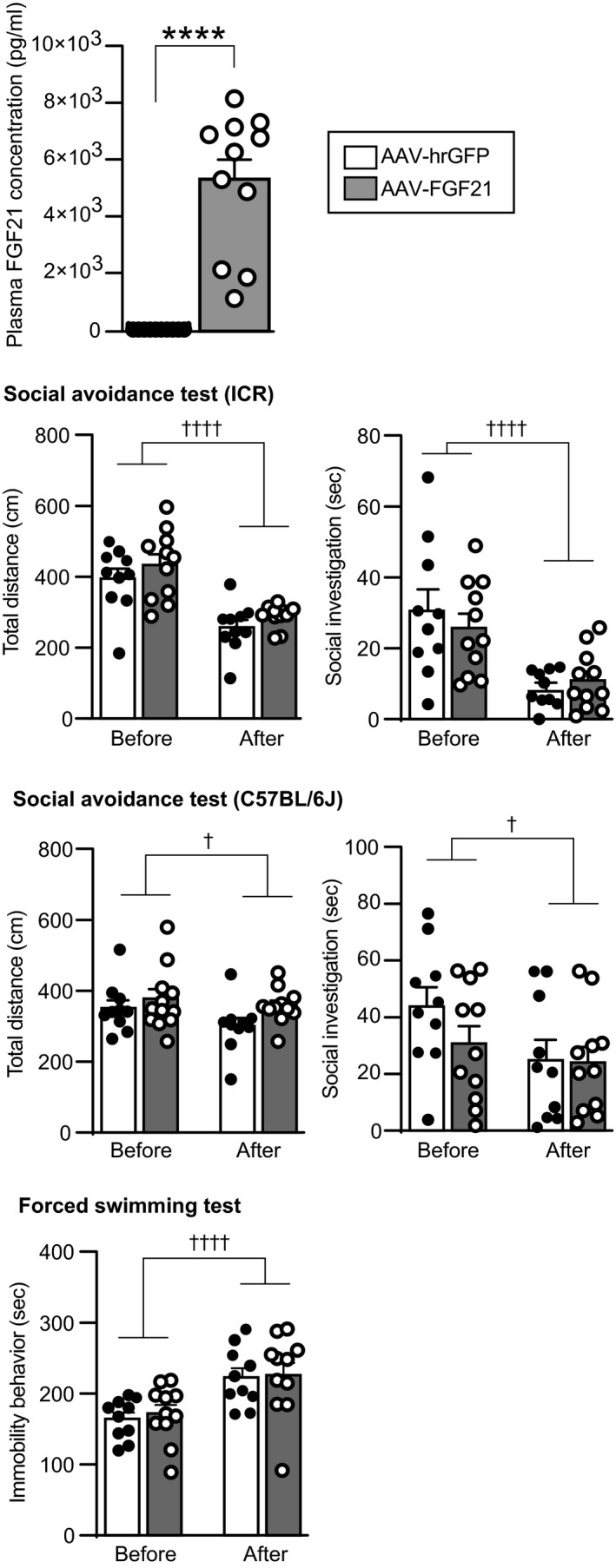
Plasma FGF21 concentrations, social avoidance behaviours and immobility behaviours in a forced swimming test in FGF21‐deficient mice injected with AAV‐hrGFP or AAV‐FGF21. Plasma concentrations of FGF21 in mice injected with AAV‐hrGFP and in mice injected with 1 × 10^9^ vg AAV‐FGF21 are shown (top). AAV‐hrGFP or AAV‐FGF21 was injected 26 days before blood sampling. Plasma FGF21 concentrations were significantly higher after injection of AAV‐FGF21 compared to after injection of AAV‐hrGFP. Total distance of locomotion and time spent for social investigation towards ICR mice in a social avoidance test are shown (second row). Total locomotor distance and time spent for investigating a cylinder containing an ICR mouse were reduced after social defeat stress, and no significant difference was found between FGF21‐deficient mice injected with AAV‐hrGFP and FGF21‐deficient mice injected with AAV‐FGF21. Total distance of locomotion and time spent for social contact with a C57BL/6J mouse in a social avoidance test are shown (third row). Total locomotor distance and time spent for investigating a cylinder containing a C57BL/6J mouse were reduced after social defeat stress, and no significant difference was found between FGF21‐deficient mice injected with AAV‐hrGFP and FGF21‐deficient mice injected with AAV‐FGF21. Duration of immobility behaviour of FGF21‐deficient mice injected with AAV‐hrGFP or AAV‐FGF21 in a forced swimming test and differences between immobility behaviour time before social defeat stress and that after social defeat stress are shown (lowest row). Time spent for immobility behaviour in a forced swimming test was reduced after social defeat stress, although no significant difference was found between mice injected with AAV‐hrGFP and mice injected with AAV‐FGF21. The number of AAV‐hrGFP‐injected and AAV‐FGF21‐injected mice was 10 and 11. ****P < 0.0001, vs AAV‐hrGFP, Mann Whitney test. †*P* < 0.05, ††††*P* < 0.0001, vs AAV‐hrGFP, two‐way repeated measures ANOVA

Plasma FGF21 concentrations in mice that had been injected with AAV‐FGF21 at 1 × 10^9^ vg/500 μL were increased compared to mice injected with AAV‐hrGFP (*U* = 0, *P* < 0.0001; Mann Whitney *U* test) (Figure 12, top).

#### 3.7.1 Social avoidance test

Locomotor activity during a social avoidance test with an ICR mouse was significantly reduced after social defeat stress compared to before social defeat stress, and no significant difference was found between mice injected with AAV‐hrGFP and mice injected with AAV‐FGF21 (no significant effect of group *F*_1,19_ = 1.851, *P* = 0.19; significant effect of time *F*_1,19_ = 45.175, *P* = 2.01 × 10^−6^; no significant interaction, *F*_1,19_ = 0.026, *P* = 0.874; repeated measures two‐way ANOVA) (Figure 12). The time spent for investigation towards the cylinder containing an ICR mouse was significantly reduced after social defeat stress compared to before social defeat stress, and no significant difference was found between mice injected with AAV‐hrGFP and mice injected with AAV‐FGF21 (no significant effect of group *F*_1,19_ = 0.097, *P* = 0.759; significant effect of time *F*_1,19_ = 29.583, *P* = 3.01 × 10^−5^; no significant interaction, *F*_1,19_ = 1.073, *P* = 0.313; repeated measures two‐way ANOVA).

Locomotor activity during a social avoidance test with a C57BL/6J mouse was significantly reduced after social defeat stress compared to before social defeat stress, and no significant difference was found between mice injected with AAV‐hrGFP and mice injected with AAV‐FGF21 (no significant effect of group *F*_1,19_ = 2.531, *P* = 0.128; significant effect of time *F*_1,19_ = 4.471, *P* = 0.048; no significant interaction, *F*_1,19_ = 0.824, *P* = 0.375; repeated measures two‐way ANOVA) (Figure 12).

The time spent for investigation towards the cylinder containing a C57BL/6J mouse was significantly reduced after social defeat stress compared to before social defeat stress, and no significant difference was found between mice injected with AAV‐hrGFP and mice injected with AAV‐FGF21 (no significant effect of group *F*_1,19_ = 0.89, *P* = 0.357; significant effect of time *F*_1,19_ = 6.483, *P* = 0.0197; no significant interaction, *F*_1,19_ = 1.483, *P* = 0.238; repeated measures two‐way ANOVA).

These findings suggest that overexpression of FGF21 did not significantly change social avoidance in FGF21‐deficient mice.

#### 3.7.2 Forced swimming test

The time spent for immobility behaviour in a forced swimming test was increased after social defeat stress compared to before social defeat stress, and no significant difference was found between mice injected with AAV‐hrGFP and mice injected with AAV‐FGF21 (no significant effect of group *F*_1,19_ = 0.099, *P* = 0.756; significant effect of time *F*_1,19_ = 40.499, *P* = 4.18 × 10^−6^; no significant interaction, *F*_1,19_ = 0.047, *P* = 0.83; repeated measures two‐way ANOVA) (Figure 12), suggesting that overexpression of FGF21 did not significantly change immobility behaviour in a forced swimming test in FGF21‐deficient mice.

## 4 DISCUSSION

The present study showed that considerable percentage of catecholaminergic neurones in the medulla oblongata and area postrema expressed β‐Klotho, the obligate coreceptor for FGF21. FGF21 activated catecholaminergic neurones in the nucleus tractus solitarius and area postrema, suggesting that noradrenergic neurones in the nucleus tractus solitarius and area postrema are targets of FGF21. Because selective destruction of noradrenergic neurones in the nucleus tractus solitarius has been reported to augment depression‐like behaviour,^23^ we examined whether FGF21 might affect depression‐like behaviour possibly via catecholaminergic neurones. In the present study, social defeat stress increased plasma FGF21 concentrations and activated catecholaminergic neurones in the medulla oblongata. FGF21‐deficient mice in the present study showed augmented depression‐like behaviour, suggesting inhibitory actions of FGF21 on depression‐like behaviour.

FGF21 acts on a receptor complex composed of an FGF receptor and β‐Klotho. β‐Klotho, which is required for high affinity binding to FGF21, is expressed in the nucleus tractus solitarius and area postrema and in the hypothalamus.^12^ In the present study, we found that administration of FGF21 activated tyrosine hydroxylase‐positive cells in the nucleus tractus solitarius and area postrema. It has been suggested that β‐Klotho in the brainstem is expressed in cells that express *Phox2b*, a homeobox gene.^12^ Phox2b has been shown to be essential for induction of the expression of dopamine β‐hydroxylase in noradrenergic neurones. We demonstrated that centrally administered FGF21 activated catecholaminergic neurones in the nucleus tractus solitarius and in the area postrema but not in the ventrolateral medulla, where no expression of β‐Klotho was found. We also demonstrated that 10%‐20% of catecholaminergic neurones express β‐Klotho and that approximately half of β‐Klotho‐expressing cells are catecholaminergic. It is thus likely that noradrenergic neurones in the nucleus tractus solitarius express β‐Klotho and that FGF21 directly activates noradrenergic neurones, although this remains to be confirmed by using triple in‐situ hybridisation for *c‐fos* mRNA, *tyrosine hydroxylase* mRNA and *β‐Klotho* mRNA. The roles of noradrenergic neurones in the nucleus tractus solitarius activated after FGF21 administration remain to be clarified. Noradrenergic neurones in the nucleus tractus solitarius project to the hypothalamus and have been shown to mediate neuroendocrine responses including activation of oxytocin^42^ and corticotrophin‐releasing hormone (CRH)^43^ neurones in response to stressful stimuli. Oxytocin and CRH have been shown to modulate various stress responses in behavioural, autonomic and metabolic systems. FGF21 has been shown to activate the hypothalamic CRH‐peripheral sympathetic nervous system, resulting in increased energy expenditure.^44^ It is tempting to speculate that activation of noradrenergic afferent projections derived from the nucleus tractus solitarius by FGF21 modulates neuroendocrine, behavioural and metabolic responses induced by social defeat stress.

Anti‐depressants have been shown to reduce the duration of immobile behaviour during a forced swimming test, whereas stressful stimuli including social defeat stress have been shown to increase the duration of immobile behaviour. Thus, the forced swimming test has been shown to enable detection of anti‐depressant actions of various compounds.^45^ On the other hand, immobile behaviour can also be interpreted as passive coping behaviour or acquired immobility rather than depression‐like behaviour during inescapable stressful stimuli^46^ and may be modulated by previous experience of a forced swimming test. Considering the present consistent data of increased immobile behaviour in a forced swimming test and augmented social avoidance in FGF21‐deficient mice, it is likely that FGF21‐deficient mice showed augmented behavioural depression‐like behaviours after social defeat stress.

A previous study showed that FGF21 administered twice per day for 3 days attenuated lipopolysaccharide‐induced depression‐like behaviour in mice.^16^ In the present study, FGF21‐deficient animals showed augmented depression‐like behaviour induced by social defeat stress, suggesting anti‐depressant actions of endogenous FGF21. On the other hand, chronic overexpression of FGF21 via peripherally administered AAV vectors in the present study had no significant effects on anxiety or depression‐like behaviours in wild‐type mice and FGF21‐deficient mice. Noradrenaline has been shown to play an important role in the development of emotional behaviours.^47^ The present study showed that noradrenergic neurones expressed β‐Klotho and that FGF21 administration activated catecholaminergic neurones. It is thus possible that FGF21 released during the developmental period has an inhibitory action on stress‐induced depression‐like behaviours via noradrenergic neurones in the nucleus tractus solitarius. Further study is necessary to determine the effects of a lack of FGF21 signalling in medullary catecholaminergic neurones during the developmental period on stress‐related behaviour in adulthood. On the other hand, FGF21 might have differential actions on behaviours dependent upon experimental conditions. Plasma FGF21 concentrations in AAV‐FGF21‐injected mice in the present study were increased to approximately 6.4 ng mL^‐1^, which is much higher than FGF21 levels after social defeat stress, although the level is comparable to or lower than FGF21 levels during starvation or ketogenic diet feeding.^12^ The effective dose of FGF21 for induction of anti‐depressant action might be low and in a narrow dose range, and overdose of FGF21 might not have anti‐depressant actions. It is also possible that exposure to AAV‐FGF21‐induced high concentrations of FGF21 for a relatively long period affected the results. Overexpression of FGF21 has been shown to induce disturbed circadian rhythm and high serum corticosterone levels.^12^ Chronic disturbed circadian rhythm and high glucocorticoid levels have been shown to exert deteriorating effects on depression status,^48^, ^49^ which might have obscured possible anti‐depressant actions of FGF21 in the present study.

The results of a previous study showed that FGF21 administration increased water intake.^41^ Consistent with those results, overexpression of FGF21 induced water intake in the present study. Endogenous FGF21 has also been shown to mediate water intake induced by alcohol ingestion or by a ketogenic diet, whereas FGF21 does not mediate basal water intake or water intake induced by dehydration.^41^ In the present study, social defeat stress‐induced water intake was not affected by FGF21 deficiency or by FGF21 overexpression, suggesting that involvement of FGF21 in the control of water intake is dependent on causes of water intake and that FGF21 does not play an important role in water intake induced by stress as in water intake induced by dehydration. In the present study, body weight was decreased after overexpression of FGF21 under the condition of social defeat stress but not under the basal condition. Both FGF21 and stress activate the hypothalamic CRH‐peripheral sympathetic system and increase energy expenditure. Considering that food intake was not changed after FGF21 overexpression or stress in the present study, it is possible that the combination of exogenous FGF21 and social defeat stress synergistically or additively increased energy expenditure, resulting in a significant decrease in body weight.

Social defeat stress has been shown to perturb lipid and glucose metabolism including reduction of fatty acid utilisation and induction of insulin resistance.^50^ Imbalanced activities of sympathetic and parasympathetic systems and increased activity of the hypothalamic‐pituitary adrenal axis observed after chronic stress may be involved in these metabolic changes.^51^ In the present study, social defeat stress transiently increased plasma concentrations of FGF21. The functions of a transient increase in FGF21 in energy metabolism remain unclear. However, FGF21 increased after stress may attenuate disturbances of lipid metabolism after chronic stress because FGF21 has been shown to increase lipid utilisation by acting on adipose tissue and on the hypothalamus^1^, ^2^ and to suppress sucrose preference,^52^ resulting in a metabolic shift from using carbohydrates to using fat.

Plasma FGF21 has been assumed to originate mainly from the liver. Peripheral FGF21 acts on peripheral organs and on the central nervous system after penetrating the blood‐brain barrier to affect energy metabolism and stress responses. Recently, production of FGF21 has also been suggested in several brain regions including the hypothalamus and hippocampus.^8^, ^16^, ^53^, ^54^, ^55^ Under certain conditions, the brain has been suggested to be the source of an increase in plasma FGF21.^55^ It remains to be determined whether social defeat stress changes FGF21 production in the brain and whether central FGF21 is responsible for anti‐depressant actions. The present study suggests that centrally administered FGF21 activates catecholaminergic neurones in the nucleus tractus solitarius, where β‐Klotho is expressed. It is tempting to speculate that these catecholaminergic neurones modulate emotion‐related behaviours.^43^ Further study is necessary to clarify whether activation of catecholaminergic neurones by centrally administered FGF21 is dependent on β‐Klotho in the nucleus tractus solitarius, or whether endogenous FGF21 activates medullary catecholaminergic neurones after social defeat stress.

In conclusion, the present study provides evidence for an anti‐depressant role of endogenous FGF21 possibly in the developmental period in mice. Considering the results showing that social defeat stress increased FGF21 in plasma, that catecholaminergic neurones expressed β‐Klotho, and that central administration of FGF21 induced activation of catecholaminergic neurones in the nucleus tractus solitarius, which have been shown to modulate energy and neuroendocrine stress responses,^43^ as well as the metabolic actions of FGF21,^12^ endogenous FGF21 systems might also play a role in the control of energy homeostasis during stress.

## AUTHOR CONTRIBUTIONS

**Naoki Usui:** Formal analysis; Investigation; Methodology; Writing – review & editing. **Masahide Yoshida:** Conceptualisation; Formal analysis; Funding acquisition; Investigation; Methodology; Resources; Supervision; Writing – review & editing. **Yuki Takayanagi:** Funding acquisition; Investigation; Methodology; Resources; Writing – review & editing. **Naranbat Nasanbuyan:** Investigation; Methodology; Writing – review & editing. **Ayumu Inutsuka:** Funding acquisition; Methodology; Writing – review & editing. **Hiroshi Kurosu:** Methodology; Resources; Writing – review & editing. **Hiroaki Mizukami:** Methodology; Resources; Writing – review & editing. **Yoshiyuki Mori:** Supervision; Writing‐review & editing. **Makoto Kuro‐o:** Conceptualisation; Methodology; Resources; Supervision; Writing – review & editing. **Tatsushi Onaka:** Conceptualisation; Funding acquisition; Investigation; Methodology; Project administration; Resources; Supervision; Writing – original draft; Writing – review & editing.

## Data Availability

The data that support the findings of this study are available from the corresponding author upon reasonable request.
